# Supplementary material for: Molecular benchmarks of a SARS-CoV-2 epidemic
Source: Nat Commun. 2021 Jun 15;12:3633. doi: 10.1038/s41467-021-23883-6 (PMC8206085; doi:10.1038/s41467-021-23883-6)
Supplement: Supplementary file 1 — Supplementary Information with tables. [file 41467_2021_23883_MOESM1_ESM.pdf]

Supplementary Information

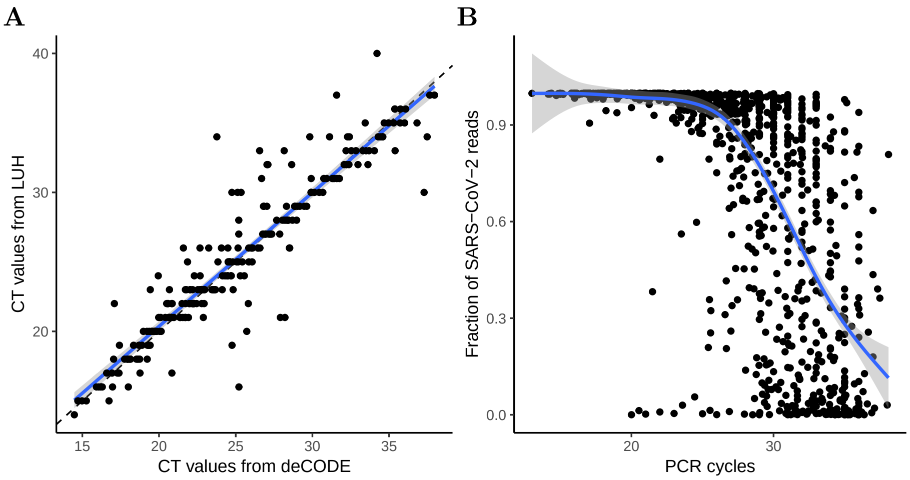

Supplementary Figure 1: Different measures of viral load.
A, CT values from LUH against CT values from deCODE. Subset of the samples (238) were measured both by LUH and deCODE. **B**, Fraction of reads mapping to SARS-CoV-2 against the aggregated CT values. The error bands are 95%-CIs and the centre in the bands are means from the regressions.

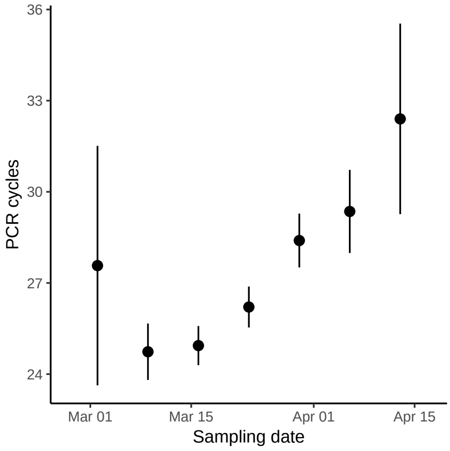

Supplementary Figure 2: qPCR-CT values as function of sampling date in the Australian data.
The centre values are means and the error bars are 95%-CIs. The number of samples in the bins are 7, 72, 252, 303, 194, 65 and 5 ordered by date.

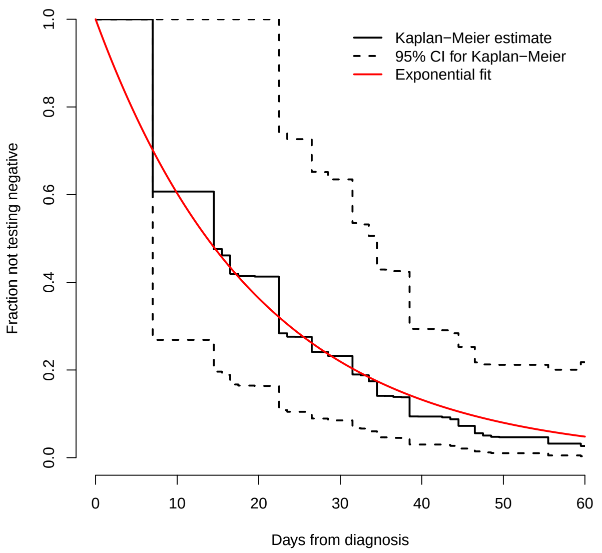

Supplementary Figure 3: Molecular traces of SARS-CoV-2 recovery with indeterminable samples. The fraction of individuals that remained positive as a function of days from the initial positive test.

A

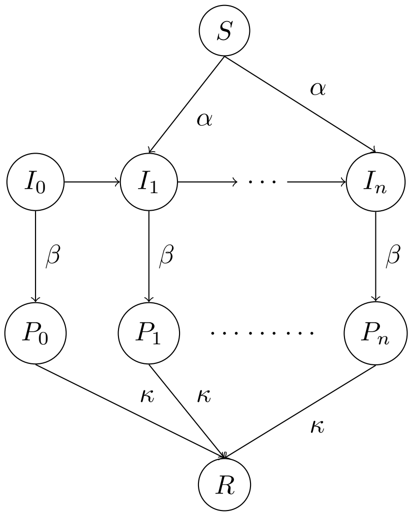

B

$$
\begin{aligned}
dS &= -\,\alpha S \sum_i I_i \\
dI_0 &= -\,\beta I_0 \\
dI_1 &= -\,\beta I_1 + \alpha S I_0 \\
&\vdots \\
dI_n &= -\,\beta I_n + \alpha S I_{n-1} \\
dP_0 &= -\,\kappa P_0 + \beta I_0 \\
&\vdots \\
dP_n &= -\,\kappa P_n + \beta I_n \\
dR &= +\,\kappa \sum_i P_i
\end{aligned}
$$

Supplementary Figure 4: Ordinary differential equations of the susceptible, infected, positive and recovered epidemiological model. **A**, schematic view of the flow of individuals in the epidemiological model. The susceptible individuals are infected by individuals in different infection generations ($I_0 \ldots I_n$). These individuals then recover from the symptoms but test positive ($P_0 \ldots P_n$). In the final stage, the positive individuals transition to the recovered and negative state. **B**, Ordinary differential equations. The infected and positive states are broken by the generation of the virus transmission to keep track of the viral generations

Supplementary Table 1: Example of a serial dilution of a positive sample

A synthetic RNA control (TaqPath control) is provided by the manufacturer at a concentration of 25 copies/µL. A serial dilution of the control shows that the detection limit in our assay setup is between approximately 1 and 6 copies of RNA. A serial dilution of a positive sample with a measured Ct value of 20.6, shows that this sample would still be determined positive after a 100,000X dilution (Ct = 35.8).

TaqPath qPCR assay			
Assay volume: 6.25 µL			
RNA input: 2.5 µL			
	Ct-value		
Sample 1	20.6		
10X dilution	23.7		
100X dilution	27.9		
1,000x dilution	31.6		
10,000X dilution	33.4		
100,000X dilution	35.8		
	Ct-value	Copies	
TaqPath-Control	30.7	62.5	
10X dilution	34.7	6.25	
100X dilution	Not determined	0.625	
