# Supplementary material for: Molecular benchmarks of a SARS-CoV-2 epidemic
Source: Nat Commun. 2021 Jun 15;12:3633. doi: 10.1038/s41467-021-23883-6 (PMC8206085; doi:10.1038/s41467-021-23883-6)
Supplement: Supplementary file 3 — Descriptions of Additional Supplementary Files [file 41467_2021_23883_MOESM3_ESM.pdf]

Descriptions of Additional Supplementary Files

Supplementary data 1
Description: Primers used for PCR amplification of viral cDNA for sequencing.

Supplementary data 2
Description: Illumina indexes used in sequencing.

Supplementary data 3
Description: The accession number of the GISAID sequences used for the comparative analysis.
