# Supplementary material for: Molecular benchmarks of a SARS-CoV-2 epidemic
Source: Nat Commun. 2021 Jun 15;12:3633. doi: 10.1038/s41467-021-23883-6 (PMC8206085; doi:10.1038/s41467-021-23883-6)
Supplement: Supplementary file 7 — Reporting Summary [file 41467_2021_23883_MOESM7_ESM.pdf]

nature research

Corresponding author(s): Hákon Jónsson

Last updated by author(s): Apr 21, 2021

Reporting Summary

Nature Research wishes to improve the reproducibility of the work that we publish. This form provides structure for consistency and transparency in reporting. For further information on Nature Research policies, see our Editorial Policies and the Editorial Policy Checklist.

Statistics

For all statistical analyses, confirm that the following items are present in the figure legend, table legend, main text, or Methods section.

n/a	Confirmed	
☐	☒	The exact sample size (*n*) for each experimental group/condition, given as a discrete number and unit of measurement
☐	☒	A statement on whether measurements were taken from distinct samples or whether the same sample was measured repeatedly
☒	☐	The statistical test(s) used AND whether they are one- or two-sided<br>*Only common tests should be described solely by name; describe more complex techniques in the Methods section.*
☐	☒	A description of all covariates tested
☐	☒	A description of any assumptions or corrections, such as tests of normality and adjustment for multiple comparisons
☐	☒	A full description of the statistical parameters including central tendency (e.g. means) or other basic estimates (e.g. regression coefficient) AND variation (e.g. standard deviation) or associated estimates of uncertainty (e.g. confidence intervals)
☐	☒	For null hypothesis testing, the test statistic (e.g. *F*, *t*, *r*) with confidence intervals, effect sizes, degrees of freedom and *P* value noted<br>*Give P values as exact values whenever suitable.*
☒	☐	For Bayesian analysis, information on the choice of priors and Markov chain Monte Carlo settings
☒	☐	For hierarchical and complex designs, identification of the appropriate level for tests and full reporting of outcomes
☐	☒	Estimates of effect sizes (e.g. Cohen's *d*, Pearson's *r*), indicating how they were calculated

Our web collection on statistics for biologists contains articles on many of the points above.

Software and code

Policy information about availability of computer code

Data collection	We used an in house developed laboratory information management system (LIMS) for tracking samples and their derived molecular extracts. The LIMS is not publicly available.
Data analysis	MCS (version 3.1) from Illumina was used for the base calling.<br>We used open source software for the sequence analysis:<br>BWA mem was used to align the short read sequences against the SARS-CoV-2 reference (https://github.com/lh3/bwa; version 0.7.16ar1181)<br>Graphtyper was used to call sequence variants and for genotyping (version 2.3.0)<br>Picard tools were used to mark duplicates (version 1.117).<br>The epidemiological model was fitted with the code in this repository https://github.com/hakon-jon/SARS-CoV-2-epi-model, in the same repository are examples demonstrating how regressions were implemented in R.

For manuscripts utilizing custom algorithms or software that are central to the research but not yet described in published literature, software must be made available to editors and reviewers. We strongly encourage code deposition in a community repository (e.g. GitHub). See the Nature Research guidelines for submitting code & software for further information.

Data

Policy information about availability of data

All manuscripts must include a data availability statement. This statement should provide the following information, where applicable:
- Accession codes, unique identifiers, or web links for publicly available datasets
- A list of figures that have associated raw data
- A description of any restrictions on data availability

We updated the data availability statement in the manuscript, listing the SARS-CoV-2 sequences and the source data behind the figures.

"
The SARS-CoV-2 sequences used in this manuscript are available at GISAID, the accession numbers are the following (with EPI_ISL prefix): 417481, 417535-417876, 424367-424624, 1585943-1585977, 1585979-1586097, 1586099-1586110, 1586112-1586121, 1586123, 1586125-1586178, 1586180-1586225, 1586227-1586267, 1586269-1586389, 1586391-1586416, 1586418-1586433, 1586435-1586438, 1586440-1586460, 1586463-1586490, 1586493-1586494, 1586496-1586571, 1586573-1586574, 1586576-1586589, 1586591-1586626, 1586628-1586636, 1586638-1586646, 1586648-1586662, 1586664-1586669, 1586671-1586777, 1586780-1586791, 1586793-1586809, 1586811-1586827, 1586829-1586846, 1586848-1586862, 1586864-1586883, 1586885-1586893. Source data behind figures are provided with this paper."

Field-specific reporting

Please select the one below that is the best fit for your research. If you are not sure, read the appropriate sections before making your selection.

[x] Life sciences   [ ] Behavioural & social sciences   [ ] Ecological, evolutionary & environmental sciences

For a reference copy of the document with all sections, see nature.com/documents/nr-reporting-summary-flat.pdf

Life sciences study design

All studies must disclose on these points even when the disclosure is negative.

Sample size	Sample size was not predetermined. This is not possible for epidemiological data from outbreaks. All positive samples at the given time in Iceland were used hence determining the sample size. Our sample size is sufficient as we determine temporal change in the viral concentration and accumulation of mutations.
Data exclusions	In the analysis of the number of mutations as function of time we excluded samples with low sequencing coverage. This was done to ensure the quality of the consensus sequence, the exclusion criteria were not pre-established.
Replication	We used viral concentration from an Australian outbreak to replicate our viral concentration findings. This is a single replication of the molecular signature of an contained epidemic in a different country and it was successful.
Randomization	Allocation is not random as the sequenced SARS-CoV-2 cases are those detected by the health care system or via the population screening of deCODE. It is not possible to randomize allocation to these two groups as individuals detected by the health care system were targeted based on symptoms, traveling from high-risk areas or history of potential exposure to infected individuals.
Blinding	The researchers were not blinded to the allocation to the groups (targeted testing and population screening) as the individuals were recruited differently.

Reporting for specific materials, systems and methods

We require information from authors about some types of materials, experimental systems and methods used in many studies. Here, indicate whether each material, system or method listed is relevant to your study. If you are not sure if a list item applies to your research, read the appropriate section before selecting a response.

Materials & experimental systems

n/a	Involved in the study	
[x]	[ ]	Antibodies
[x]	[ ]	Eukaryotic cell lines
[x]	[ ]	Palaeontology and archaeology
[x]	[ ]	Animals and other organisms
[ ]	[x]	Human research participants
[x]	[ ]	Clinical data
[x]	[ ]	Dual use research of concern

Methods

n/a	Involved in the study	
[x]	[ ]	ChIP-seq
[x]	[ ]	Flow cytometry
[x]	[ ]	MRI-based neuroimaging

Human research participants

Policy information about studies involving human research participants

Population characteristics	The individuals diagnosed by the health care system are those reporting symptoms, traveling from high-risk areas or have history of potential exposure to infected individuals. Individuals diagnosed by the population screening were asymptotic or with mild symptoms. The population screening was carried out in the capital area of Iceland. The average age of positive individuals was 40.7 years and 49.7% of the positive individuals were males.
Recruitment	Individuals were diagnosed through the health care system and through the population screening. The number of individuals in each group are stated in the main text. The individuals diagnosed by the health care system were those reporting symptoms, traveling from high-risk areas or have history of potential exposure to infected individuals. The population screening was done in two ways: first the registrations to the test was open to all residents of Iceland on line, secondly we sent an invitation through text message to a random subset of Icelanders between ages 20 and 70 years. Individuals participating in the population screening could be worried about their infection status due to mild symptoms and hence could have a higher/lower viral concentration compared to the general public.
Ethics oversight	The study was approved by the National Bioethics Committee of Iceland (Approval no. VSN-20-070).

Note that full information on the approval of the study protocol must also be provided in the manuscript.
